# Elucidating the Mechanism of Oridonin in Treating Hepatocellular Carcinoma: Network Pharmacology and Experimental Validation

**DOI:** 10.1111/jcmm.71180

**Published:** 2026-06-03

**Authors:** Long Li, Xiaodi Guo, Gangqiang Wang, Jing Zhang, Jin Wang, Shan Miao, Yang Sun, Shanbo Ma, Xiaopeng Shi

**Affiliations:** ^1^ Department of Pharmacy, Xijing Hospital Fourth Military Medical University Xi'an China; ^2^ Department of Clinical Laboratory, Xijing Hospital Fourth Military Medical University Xi'an China; ^3^ Key Laboratory of Gastrointestinal Pharmacology of Chinese Materia Medica of the State Administration of Traditional Chinese Medicine Fourth Military Medical University Xi'an China

**Keywords:** dynamics simulation, EGFR/PI3K/AKT, hepatocellular carcinoma, molecular docking, network pharmacology, oridonin

## Abstract

Oridonin, a bioactive diterpenoid derived from Rabdosia rubescens, has significant anti‐tumour activity. Although previous studies have shown that oridonin has anti‐liver cancer potential, the molecular mechanism of its treatment of hepatocellular carcinoma (HCC) still needs to be further investigated. This study utilized network pharmacology, molecular docking, and molecular dynamics simulations (MDS) to elucidate the mechanisms by which oridonin exerts its therapeutic effects on HCC. The findings were then validated by in vitro experiments. Initially, potential targets of oridonin for HCC were identified through online database retrieval. The mechanisms of oridonin in resisting HCC were then elucidated via integrated protein–protein interaction (PPI) network analysis, GO and KEGG enrichment analysis. Subsequently, these findings were confirmed through molecular docking, MDS and the in vitro experiments. A total of 273 intersecting targets were identified. Subsequently, PPI network analysis was performed, leading to the identification of 10 core targets. Molecular docking studies between these targets and oridonin suggested that PIK3R1, EGFR, AKT1, and JAK2 might be key targets. Further MDS demonstrated a strong interaction between oridonin and these key targets. Subsequent in vitro experiments revealed that oridonin significantly affects the cell cycle and induces apoptosis in HCC cells. Furthermore, oridonin significantly inhibits the expression of key targets within the EGFR/PI3K/AKT signalling pathway. These findings suggest that oridonin may exert anti‐cancer effects through the EGFR/PI3K/AKT signalling pathway, providing a robust theoretical foundation for its clinical application and mechanistic investigation in HCC treatment.

## 1 Introduction

According to the 2020 global cancer data statistics, primary liver cancer is the sixth most prevalent cancer globally and constitutes the third leading cause of cancer‐related mortality [1]. Hepatocellular carcinoma (HCC) accounts for over 80% of primary liver cancer instances, presenting a substantial global public health concern [2]. The primary treatment modalities for HCC currently include surgical removal, organ transplant, ablation, transarterial chemoembolization (TACE), and drug therapy [3, 4]. Despite the availability of these treatment modalities that can yield certain therapeutic benefits, the overall efficacy and prognosis for HCC remain suboptimal, characterised by significant side effects and high financial costs [5, 6]. Patients in advanced stages of the disease frequently experience deteriorating liver function, thereby restricting the multiple treatments of TACE. Furthermore, HCC exhibits inherent resistance to chemotherapy, resulting in diminished anti‐tumour efficacy of chemotherapeutic agents, negligible improvements in overall survival, and significant associated toxicities [7, 8]. Consequently, the pursuit of novel therapeutics with targeted efficacy and reduced adverse effects has become a prominent area of research in HCC treatment [9].

Traditional Chinese Medicine (TCM) has been widely recognized for its safety, efficacy, low toxicity, and moderate price in tumour treatment during clinical practice, making it a hot topic in HCC research [10, 11]. Clinical research has demonstrated that TCM contributes significantly to the comprehensive treatment of HCC by enhancing therapeutic efficacy, alleviating symptoms, reducing toxicity, decreasing tumour recurrence and metastasis, extending patient survival, and improving quality of life [12, 13, 14]. Furthermore, numerous bioactive compounds found in Chinese herbal medicine exhibit potent anti‐cancer properties, including alkaloids, flavonoids, and terpenoids [15, 16, 17, 18]. Oridonin, a bioactive diterpenoid sourced from Rabdosia rubescens, exhibits a range of pharmacological effects, including anti‐inflammatory [19], anti‐neoplastic [20], antibacterial [21], anti‐sepsis [22], neuroprotection [23], and immune regulation [24]. Its anti‐cancer properties have been proven in a variety of vicious tumours, including gastric, colorectal, oesophageal, breast, and ovarian tumours, among others [25, 26]. Recent studies have demonstrated that oridonin can synergistically augment the therapeutic efficacy of anticancer drugs [27, 28]. However, the precise mechanisms underlying oridonin's anti‐HCC effects remain inadequately elucidated, necessitating further comprehensive investigation.

Network pharmacology offers a novel avenue for drug research by employing a multi‐targeted approach [29]. By conducting an in‐depth examination and amalgamation of extensive datasets, a more comprehensive prediction of potential drug targets and signalling pathways can be achieved [30]. Molecular docking, a computer‐assisted drug design technique that relies on the interaction and affinity between targets and active compounds, has found widespread application in elucidating the pharmacological basis of traditional Chinese medicine [31]. Molecular dynamics simulation (MDS) is instrumental in validating the stability of the interactions between active components and key targets [32]. The study provided a thorough analysis of the potential molecular targets and signalling pathways involved in treating HCC with oridonin. Subsequently, the aforementioned findings were validated through molecular docking, MDS, and molecular biology experiments to provide a deeper understanding of how oridonin functions in the HCC therapy.

## 2 Materials and Methods

### 2.1 Prediction of Potential Targets of Oridonin

Obtained the chemical structure of oridonin from the PubChem database, then imported it into Pharm Mapper (https://lilab‐ecust.cn/pharmmapper/submitfile.html) and Herb (http://herb.ac.cn/) data for search, set the category to *Homo Sapiens* , and obtained the targets of oridonin.

### 2.2 Prediction of Targets for HCC

HCC gene expression profiles data were obtained from the GEO database (https://portal.gdc.cancer.gov/). Only datasets with over 20 samples that included both tumour and normal tissue samples were considered. GSE29721, GSE45267, and GSE112790 met these conditions. Differential analysis was performed utilizing the limma package in R software and integrated with the RobustRankAgggreg package for visualization using ggplot2. Furthermore, targets of HCC were identified by searching DrugBank, TTD, OMIM, and Gene Cards databases with “Hepatocellular carcinoma” as the keyword. These targets were integrated for further analysis.

### 2.3 Constructing the Common Target of “Drug‐Disease”

The venn diagram was generated using the Venn online tool (https //bioinfogp.cnb.csic.es/tools/venny/) to compare oridonin‐related targets with disease targets. STRING was then used to upload the overlap targets, with *Homo sapiens* selected as the species and a high confidence level set to 0.9. Subsequently, a protein interaction network diagram was obtained.

### 2.4 Core Target Screening and Enrichment Analysis

The results of protein–protein interaction were brought into Cytoscape 3.9.1 for network analysis and visualization, and the first 10 core targets were screened by cytohubba plug‐in. Using the GO database and R, functional enrichment analysis was conducted on the intersection genes for CC, MF, and BP. Additionally, enrichment analysis using KEGG was carried out on the shared targets, with the top 30 enriched results being visualized.

### 2.5 Molecular Docking

The core active ingredient of the drug was subjected to molecular docking with the core target to confirm the bonding affinity. The compound structure file in “sdf” format was acquired from PubChem. AutoDockTools 1.5.6 was used to conduct hydrogenation calculations on the three‐dimensional structure, resulting in the generation of a “pdbqt” file. The primary target protein's three‐dimensional structure was obtained from the Protein Data Bank (PDB) database, saved as a “pdb” file, dehydrated, and hydrogenated with AutoDockTools 1.5.6. The Grid module was employed to define the proprotein ligand as the centre of the docking box and to establish interaction regions for the molecules. Subsequently, Autodock Vina molecular simulation software was employed to examine the docking between the compounds and target proteins, confirming the outcomes of network pharmacology.

### 2.6 Molecular Dynamics Simulation

The software Desmond/Maestro version 2022.1, which is noncommercial, was chosen as the dynamics simulation software for conducting MDS on a protein‐ligand complex to investigate ligand stability. The TIP3P water model was utilized to incorporate water molecules into the system, along with the addition of a 0.15 M sodium chloride solution for system balance. After reducing the system and allowing it to relax, MDS were conducted for 100 ns at 300 Kelvin and 1 atm pressure with constant temperature and pressure. Coordinate tracking was performed every 100 ps. The Desmond simulation interaction diagram was used for molecular dynamics analysis, with GROMACS being used to determine the Gibbs relative free energy using Root Mean Square Deviation (RMSD) and Radius of gyration (Rg) values. Additionally, the free energy landscape was visualized using RMSD, Rg, and Gibbs relative free energy values.

### 2.7 Cell Culture

HepG2 and Huh7 from the Cell Bank at the Chinese Academy of Sciences in Shanghai, China are the two most widely used HCC cell lines with distinct genetic backgrounds (HepG2 harbours wild‐type TP53, while Huh7 has a TP53 mutation (Y220C); HepG2 exhibits low metastatic potential, while Huh7 displays higher metastatic capacity and stronger proliferative ability), which allows us to verify the anti‐HCC effect of oridonin across different genetic contexts.

Both cell lines were authenticated by Short Tandem Repeat (STR) profiling (performed by Biowing Biotechnology, Shanghai, China) before the experiment. The STR profiles matched the standard of DSMZ databases (GS‐HepG2 (RCB1681) and HuH‐7 (JCRB0403)) confirm no cross contamination. The test numbers are 20,240,527–01 and120240612‐03.

The cells were cultured in Dulbecco's modified Eagle's medium (DMEM) supplemented with 10% fetal bovine serum, 1% penicillin–streptomycin at 37°C with 5% CO_2_.

### 2.8 Drugs

Accurately measure the oridonin powder and melt it in dimethyl sulfoxide (DMSO) to create a stock solution with a concentration of 10 mM. The solution should then be processed through a 0.22 μm microporous filter and stored at 4°C for light preservation. When using, dilute with culture medium as needed.

### 2.9 Cell Viability

Cells in exponential growth phases of HepG2 and Huh7 were placed at a density of 5*10^3^ cells/well in a 96‐well plate and incubated for 24 h. The oridonin stock solution was diluted to various concentrations (0.5–40 μM) and added to the plate. Six duplicate wells were set up for each concentration and incubated at 37°C with 5% CO_2_ for 24 and 48 h, respectively. Following incubation, 10 μL of Cell counting Kit‐8 (CCK8) solution (Solebao, Beijing, China) was included in each well and incubated for an additional 2 h. The plate was then removed from the incubator, and the absorption at 450 nm was measured with a microplate reader.

### 2.10 Detection of Cell Cycle and Apoptosis Using Flow Cytometry

HepG2 and Huh7 cells were harvested and seeded in six‐well plates during a phase of logarithmic growth. Upon reaching 60% to 70% confluency, oridonin was introduced. The experiment included a control group without drug treatment, as well as low‐ and high‐dose experimental groups, each with 3 replicates. After 24 h, the cells were harvested, rinsed twice with 1 mL of cold phosphate‐buffered saline (PBS).

Cell cycle analysis: the cells were re‐suspended in 1 mL of 70% ethanol pre‐chilled at 4°C, then fixed at 4°C for 2 h. Then, each sample was supplemented with 0.5 mL of propidium iodide (PI) staining solution, thoroughly mixed, and incubated at 37°C in the dark for 30 min. In conclusion, the samples were assessed through the use of flow cytometry.

Cell apoptosis: the cells were placed in 195 μL of Annexin V‐FITC binding solution, along with 5 μL of Annexin V‐FITC and 10 μL of PI staining solution, and were gently agitated. Afterward, the cells were left to incubate at room temperature without light for a duration of 10 to 20 min, following which flow cytometry was used to assess cellular apoptosis.

### 2.11 Immunofluorescence

HepG2 and Huh7 cells in the logarithmic growth phase were placed in a 24‐well plate with chamber slides at a concentration of 2 × 10^4^ cells per well, and incubated in 1 mL of medium for 24 h. Following removal of the culture medium, the normal group received complete medium whereas the experimental group was exposed to full medium with different levels of oridonin. The expression level of p‐AKT was assessed 24 h post‐treatment.

### 2.12 qRT‐PCR

Trizol protocol was used for RNA extraction, followed by reverse transcription and real‐time PCR analysis following kit guidelines. The reverse transcription step involved incubation at 25°C for 5 min, 42°C for 30 min, 85°C for 5 min, and subsequent storage at 4°C. PCR parameters consisted of an initial heating at 95°C for half a minute, then heating at 95°C for 5 s, and finally cooling at 60°C for 30 s for a total of 40 cycles. The melting curve program consisted of steps at 95°C for 15 s, 60°C for 60 s, and 95°C for 15 s. Details of the qPCR primer sequences were available in Table 2. Gene sequences were acquired from the National Center for Biotechnology Information. The reference gene used was GAPDH, and data analysis was carried out through the 2^−△△Ct^ method.

### 2.13 Western Blotting

Protein extracts were prepared from cells utilizing radioimmuno‐precipitation assay lysis buffer (Beyotime, Shanghai, China) supplemented with protease and phosphatase inhibitors. The protein concentration was quantified using the bicinchoninic acid kit (Beyotime). Subsequently, proteins (30 μg per lane) were resolved by 10% SDS‐PAGE (Beyotime) and transferred onto a PVDF membrane. The membrane was blocked with 5% nonfat milk for 2 h and then incubated overnight at 4°C with the primary antibody, followed by a 1‐h incubation with the secondary antibody (1:2000, Abcam, Cambridge, UK). Protein bands were visualized using enhanced chemiluminescence reagent (Millipore, Billerica, MA). Protein expression levels were quantified using ImageJ software, with GAPDH serving as the loading control. The antibodies used were as follows: EGFR (ab52894, Abcam, UK), PI3K (ab151549, Abcam), AKT (ab8805, Abcam), and GAPDH (AF7021, Affinity, USA).

### 2.14 Statistical Analyses

Each experiment was conducted independently, with a minimum of three repetitions, and the findings were reported in the form of mean ± standard deviation (SD) and were analysed using One‐way ANOVA, with a significance level of *p* < 0.05.

## 3 Results

### 3.1 Analysis of Possible Targets of Oridonin

**FIGURE 1:**
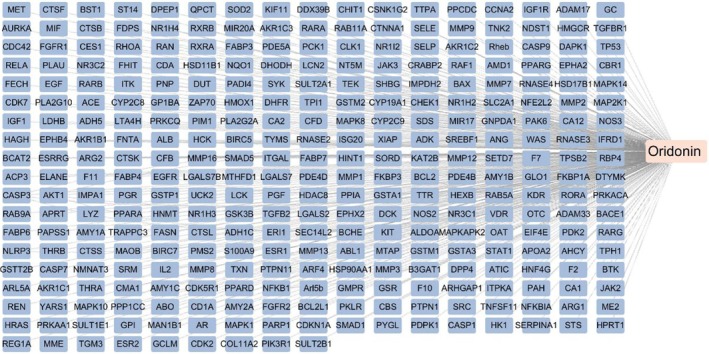
Potential targets of oridonin.

A total of 315 potential targets were identified in the Pharm mapper database, while 31 potential targets were identified in the Herb database. The intersection of these findings has identified 315 potential targets for further investigation (Figure 1).

### 3.2 Target Prediction of HCC

**FIGURE 2:**
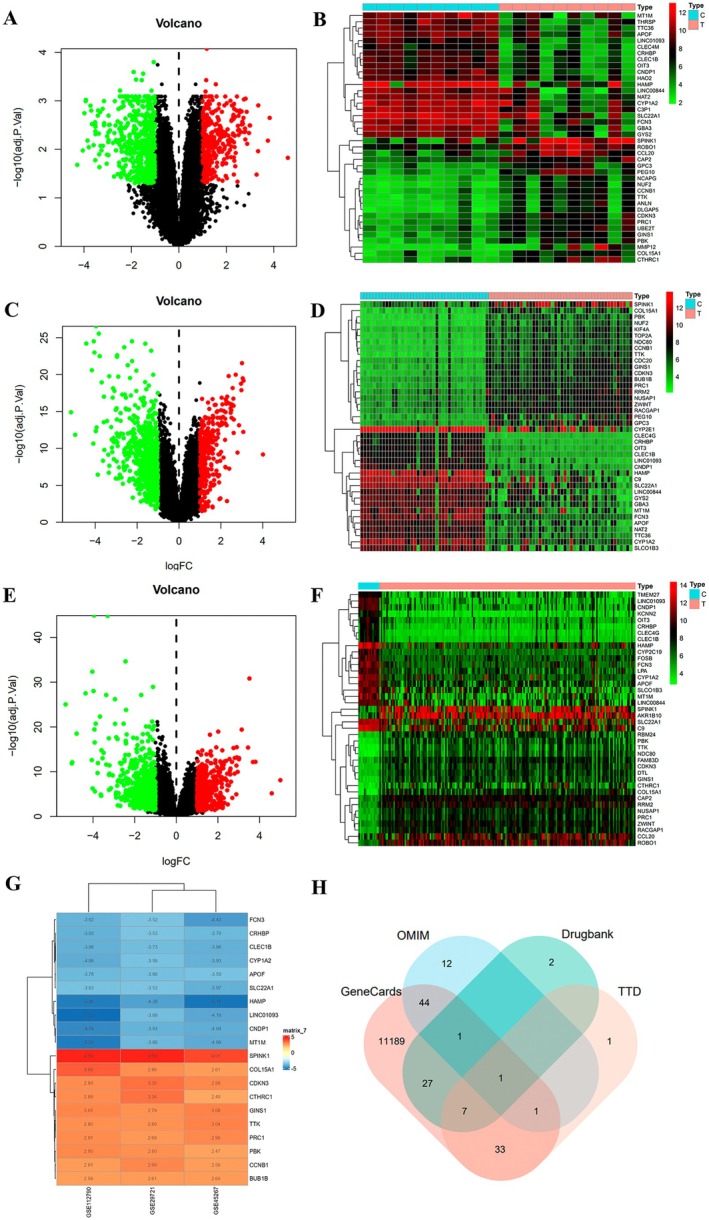
(A, C, E) Volcano maps of DEGs in GSE29721, GSE45267, GSE112790. (B, D, F) Heat maps of GSE29721, GSE45267, GSE112790. (G) Heat map of top 20 up‐regulated and down‐regulated DEGs. (H) HCC‐related targets of disease databases.

Gene differential analysis was performed by employing the Limma package on data from the Gene Expression Omnibus (GEO) database and visualized with ggplot2. Within the GSE29721 dataset, there were 821 genes that exhibited varying levels of expression, including 460 genes that were increased and 361 genes that were decreased (Figure 2A,B). There were 941 genes found to be expressed differently, with 900 genes showing an increase in expression and 41 genes showing a decrease for the GSE45267 dataset (Figure 2C,D). In the GSE112790 dataset, 1027 genes showed variations in expression, with 865 genes up‐regulated and 162 genes down‐regulated (Figure 2E,F). Integration of the differential genes using Robust Rank Aggregation (RRA) revealed 207 differential genes, consisting of 84 up‐regulated and 123 down‐regulated genes (Figure 2G). Disease database searches yielded 11,303 related targets (Relevance score ≥ 1) in the Gene Cards database, 177 related targets in the Online Mendelian Inheritance in Man (OMIM) database, and 42 correlated targets in the DrugBank database. Additionally, 43 related targets were identified in the Therapeutic Target Database (TTD) database, resulting in a total of 11,318 HCC targets after removing duplicates (Figure 2H).

### 3.3 Enrichment Analysis

**FIGURE 3:**
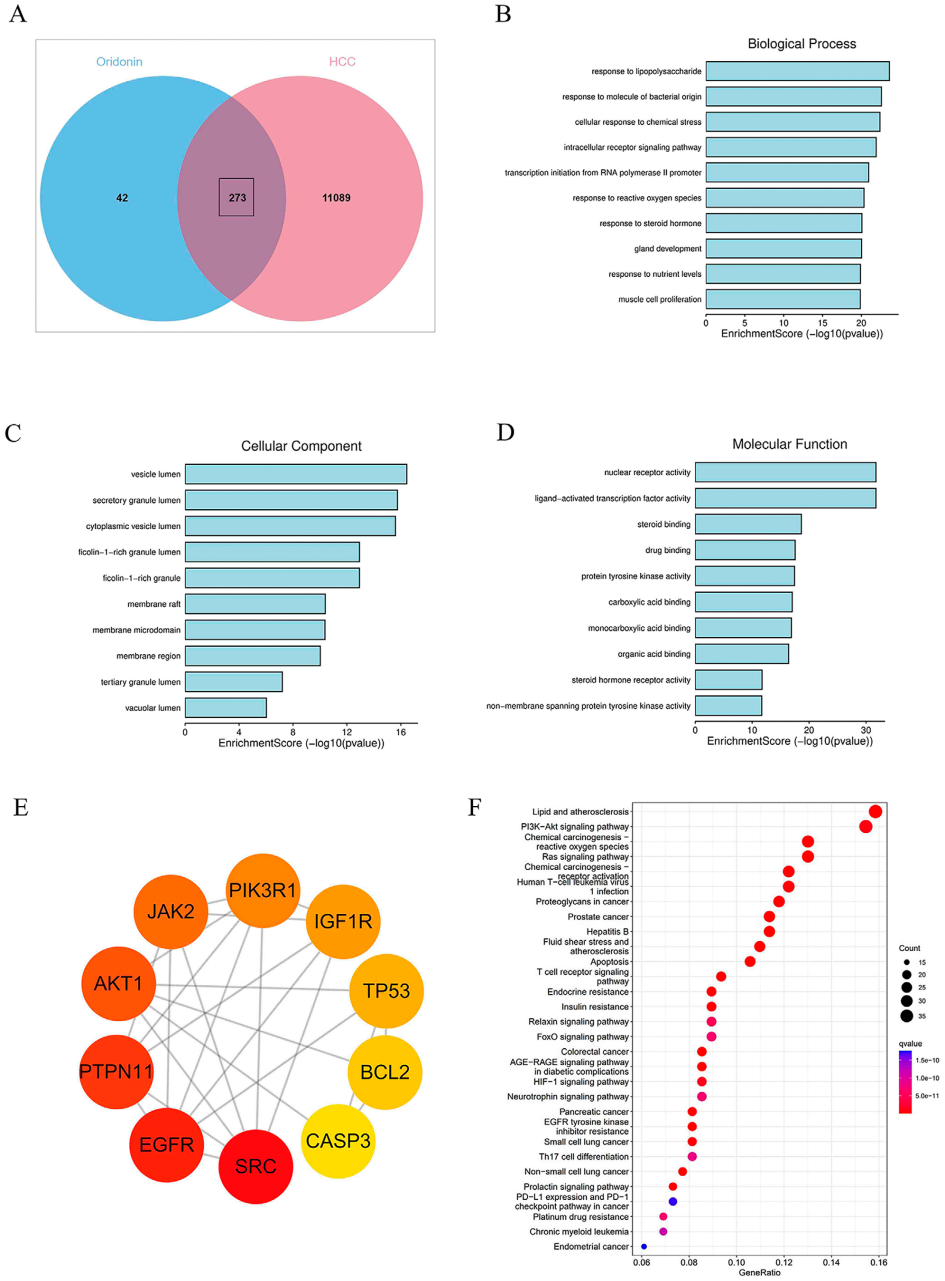
(A) Venn diagram of oridonin and HCC. (B) PPI network. (C–E) BP, CC, MF in GO enrichment analysis. (F) Top 10 core targets. (G) KEGG enrichment analysis.

A comparison was made between the disease and drug targets, leading to a sum of 273 common targets (Figure 3A). The shared targets were then entered into the STRING database, using *Homo sapiens* as the species and setting high confidence to 0.9, to create a protein–protein interaction (PPI) network (Figure 3B). The Cytohub plugin was utilized to filter the top 10 ranked targets, which included SRC, EGFR, PTPN11, AKT1, JAK2, PIK3R1, IGF1R, TP53, BCL2, and CASP3 (Figure 3F). Additionally, the Gene Ontology (GO) and the Kyoto Encyclopedia of Genes and Genomes (KEGG) were performed on the overlapping targets (*p* < 0.05). The GO enrichment analysis revealed involvement in biological processes (BP) such as cell response to chemical stress, intracellular receptor signalling pathway, and cell response to reactive oxygen species, among others. Cellular components (CC) included membrane rafts, membrane microstructures, and vesicular lumen, while molecular functions (MF) included activity of nuclear receptors, activity of transcription factors activated by ligands, and more (Figure 3C–E). Furthermore, KEGG enrichment analysis showed significant enrichment in lipid metabolism, atherosclerosis, and the PI3K‐AKT signalling pathway, with a greater number of core targets enriched in the latter pathway (Figure 3G).

### 3.4 Molecular Docking

**FIGURE 4:**
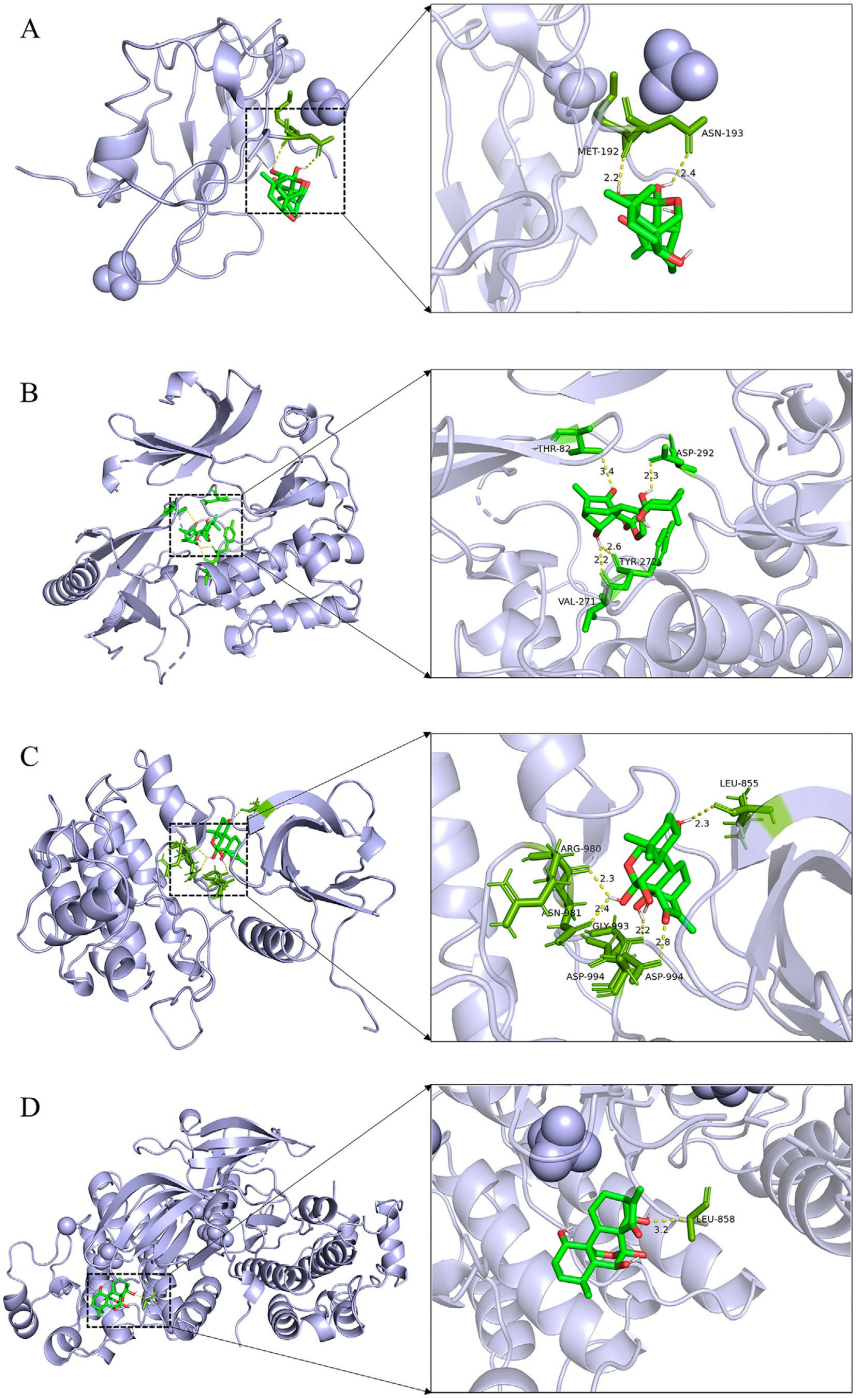
Three‐dimensional binding mode. (A) PIK3R1‐Oridonin. (B) AKT1‐Oridonin. (C) JAK2‐Oridonin. (D) EGFR‐Oridonin.

In order to investigate the interaction between oridonin and the hub gene, oridonin (CAS: 28957–04‐2) was utilized as the ligand, while the crystal structure of the core targets was acquired from the PDB as the receptor. It is widely acknowledged that a binding energy of ≤ −5 kcal/mol indicates a high affinity between small molecules and proteins [33]. The binding energies of oridonin with core target proteins, as depicted in Table 1, all fell below −7 kcal/mol, signifying excellent binding affinity. The top 4 docking results were visualized using PyMOL. Oridonin interacted with PIK3R1 via amino acid residues MET‐194 and ASN‐193, forming hydrogen bonds. Similarly, it engaged with AKT1 through amino acid residues ASP‐292, THR‐82, TYR‐272, and VAL‐271, establishing four hydrogen bonds. Oridonin also established four hydrogen bonds with JAK2 at ASP‐994, GLY‐993, ASN‐981, ARG‐980, and LEU‐855; and with EGFR, it formed a 3.2 Å hydrogen bond at LEU‐858 (Figure 4).

**TABLE 1:** Free energy of binding.

Ligand	Receptor	Free energy of binding (kcal/mol)
Oridonin	PIK3R1	−9.2
Oridonin	AKT1	−9.2
Oridonin	JAK2	−8.9
Oridonin	EGFR	−8.4
Oridonin	SRC	−8.2
Oridonin	IGF1R	−8.1
Oridonin	BCL2	−7.7
Oridonin	TP53	−7.6
Oridonin	CASP3	−7.4
Oridonin	PTPN11	−7.2

### 3.5 Molecular Dynamics Simulation

To assess the stability of protein‐ligand complexes in dynamic systems, MDS was conducted. While molecular docking reveals potential interactions between the ligand and protein, MDS could quantify the strength and duration of these interactions.

#### 3.5.1 RMSD

**FIGURE 5:**
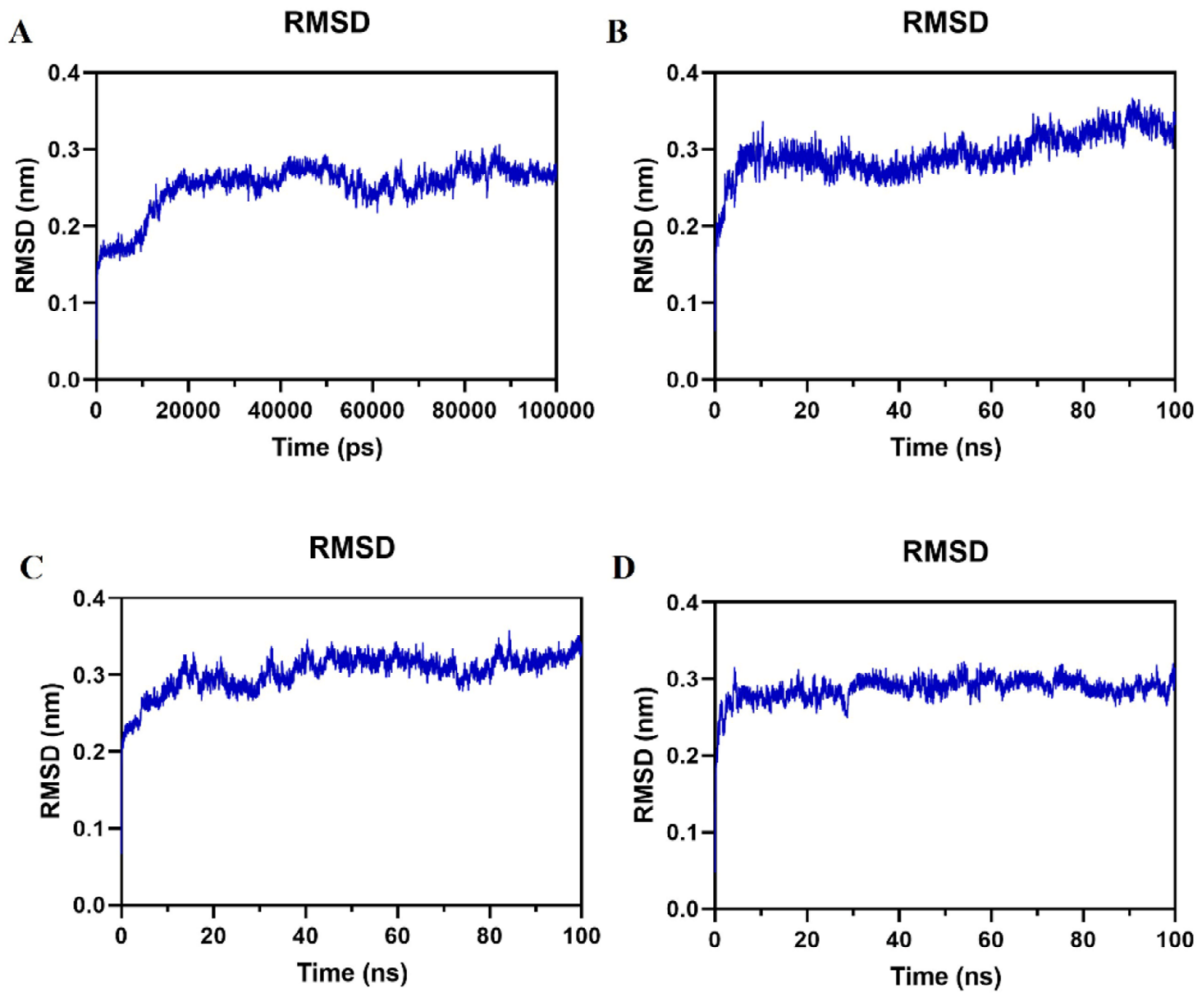
RMSD plots (A) PIK3R1‐Oridonin. (B) AKT1‐Oridonin. (C) JAK2‐Oridonin. (D) EGFR‐Oridonin.

Throughout the 100 ns simulation, the complexes displayed significant fluctuations in the first few nanoseconds before stabilizing around 0.3 nm, indicating good stability (Figure 5).

#### 3.5.2 Root Mean Square Fluctuation (RMSF)

**FIGURE 6:**
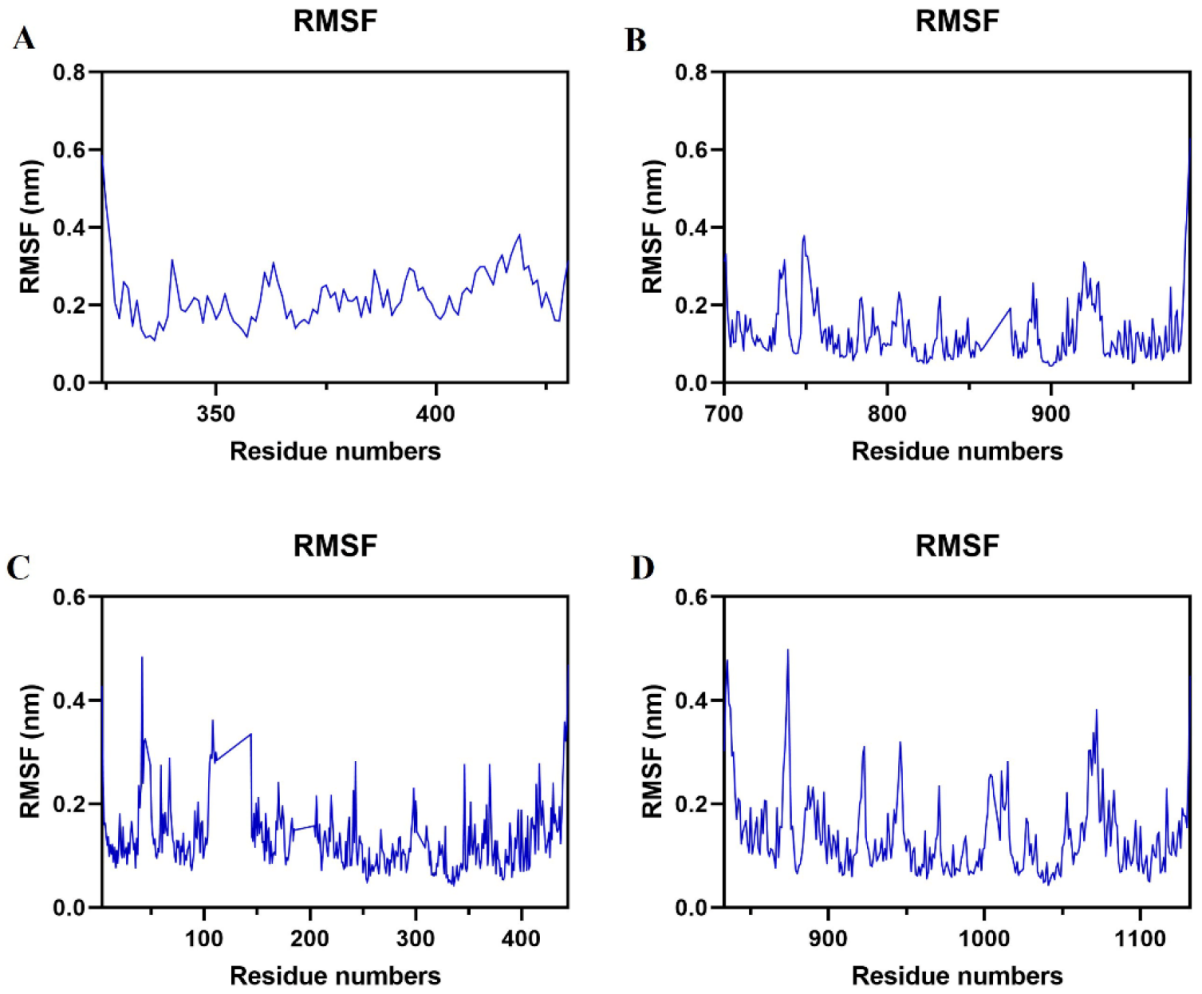
RMSF plots (A) PIK3R1‐Oridonin. (B) AKT1‐Oridonin. (C) JAK2‐Oridonin. (D) EGFR‐Oridonin.

RMSF analysis can be utilized to assess the fluctuation levels of individual amino acid residues when proteins interact with ligands during simulation. The complexes consistently demonstrated low RMSF values, suggesting stable protein‐ligand interactions. During equilibrium fluctuations, loop or terminal residues typically exhibited higher RMSF values (Figure 6).

#### 3.5.3 Hydrogen Bonds

**FIGURE 7:**
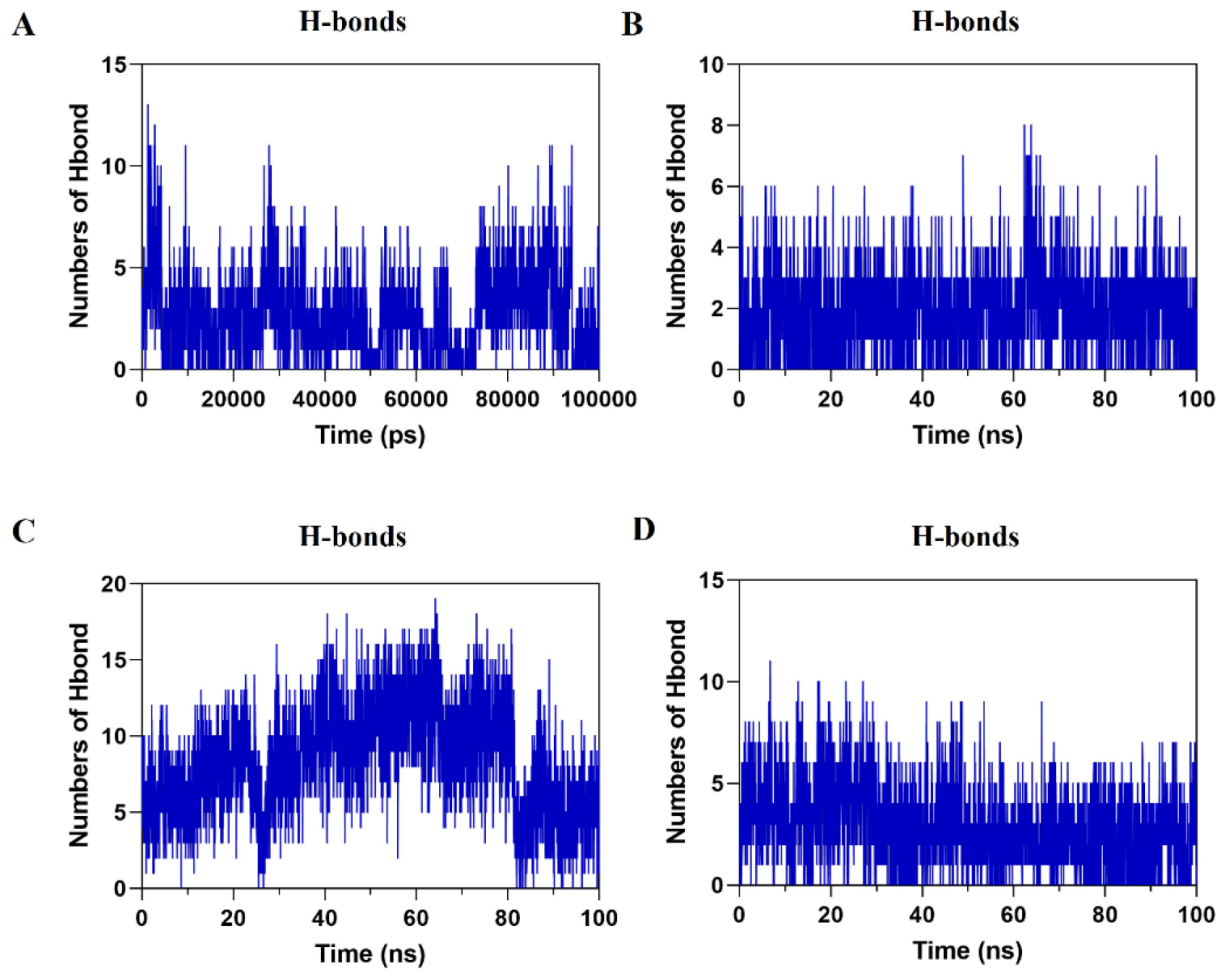
Hydrogen bonds (A) PIK3R1‐Oridonin. (B) AKT1‐Oridonin. (C) JAK2‐Oridonin. (D) EGFR‐Oridonin.

Hydrogen bonds play a vital role in maintaining the stability of protein‐ligand interactions. The analysis revealed that the oridonin‐JAK2 complex formed the most hydrogen bonds, exceeding 15 during the 100 ns simulation. Furthermore, the oridonin‐PIK3R1 and oridonin‐EGFR complexes formed over 10 hydrogen bonds each during the simulation period, while the number of hydrogen bonds of the oridonin‐AKT1 complex remained relatively stable, ranging between 3 and 6 (Figure 7).

#### 3.5.4 Radius of Gyration (Rg)

**FIGURE 8:**
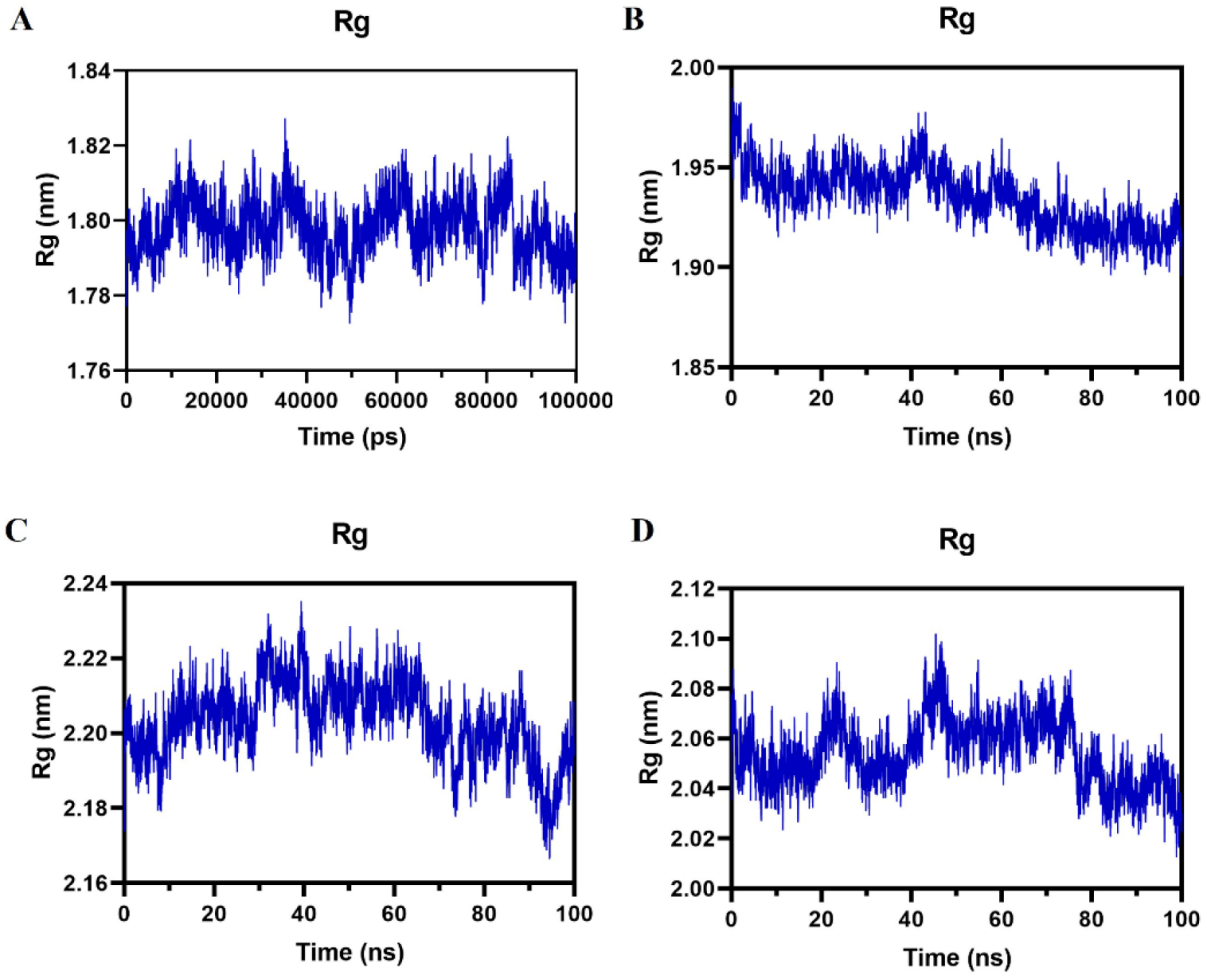
Rg plots (A) PIK3R1‐Oridonin. (B) AKT1‐Oridonin. (C) JAK2‐Oridonin. (D) EGFR‐Oridonin.

The Rg reflects the compactness and folding stability of the protein‐ligand binding process. A lower Rg value indicates a higher system density and tighter arrangement. Analysis of the Rg values for complexes, as depicted in Figure 8, revealed fluctuations within specific ranges: 1.78–1.82 nm for the oridonin‐PIK3R1 complex, 1.90–1.95 nm for the oridonin‐AKT1 complex, 2.18–2.22 nm for the oridonin‐JAK2 complex, and 2.03–2.10 nm for the oridonin‐EGFR complex. These findings suggested that all of these complexes exhibited a relatively compact structure.

#### 3.5.5 Free Energy Landscape (FEL)

**FIGURE 9:**
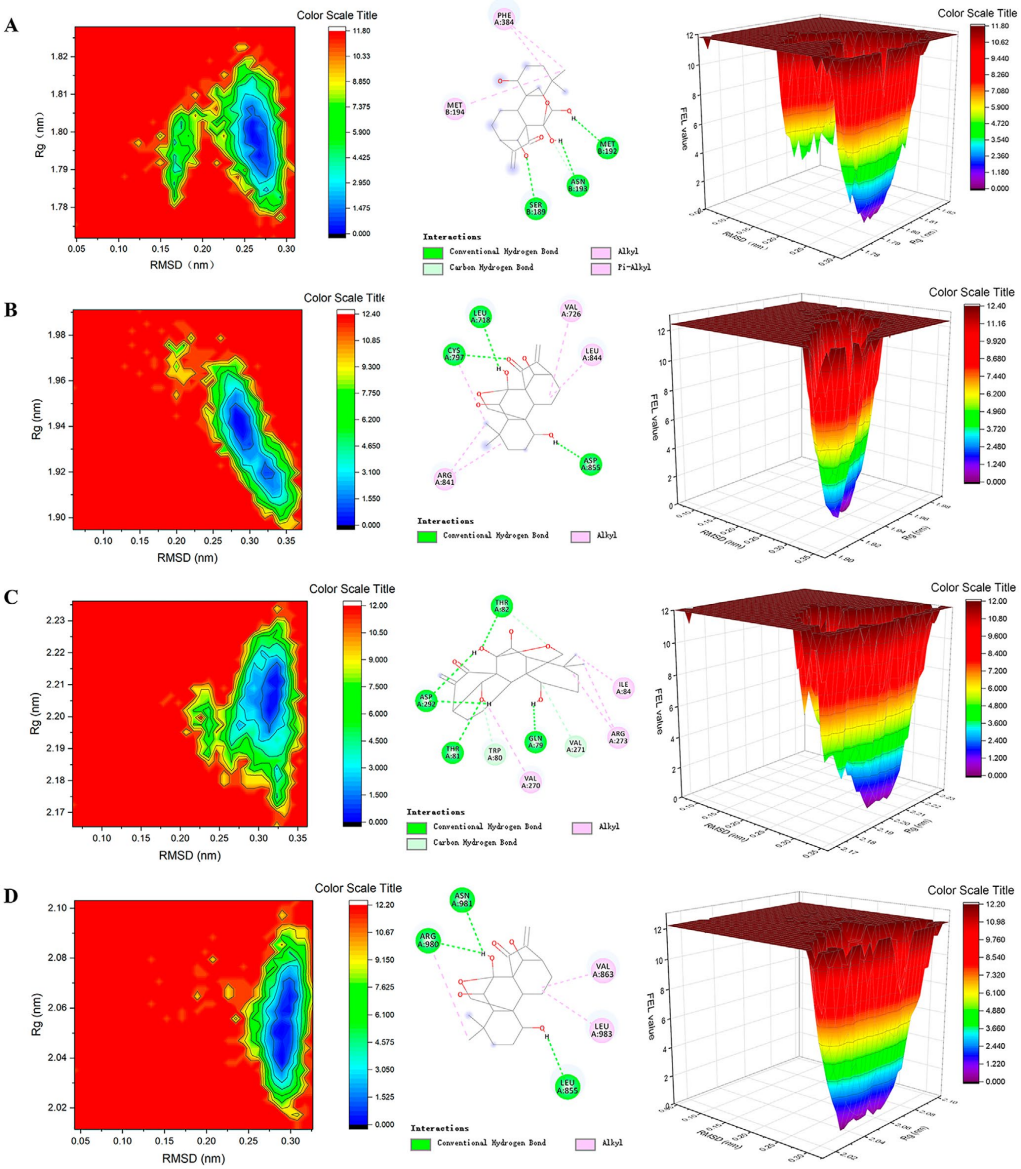
FEL plots (A) PIK3R1‐Oridonin. (B) AKT1‐Oridonin. (C) JAK2‐Oridonin. (D) EGFR‐Oridonin.

The conformations obtained from MDS can be used to analyse the changes in free energy experienced by the material throughout the simulation process. Each protein‐ligand complex exhibits a distinct pattern in the FEL. Deep blue spots indicate energy minima and energetically favourable protein conformations, while yellow‐green spots represent unfavourable conformations. The PIK3R1‐oridonin complex was considered relatively stable when the Rg value fell within the range of 1.785 to 1.81 nm and the RMSD value was between 0.25 and 0.3 nm. During this stable conformational state, the complex engaged in hydrogen bond interactions with amino acid residues MET B: 192, ASN B: 193, and SER B: 189, along with three Pi‐Alkyl interactions involving PHE A: 384 and MET B: 194 (Figure 9A). Similarly, for the AKT1‐oridonin complex to be stable, the Rg value should be in the range of 1.91 to 1.95 nm and the RMSD value between 0.25 and 0.35 nm. In this state, the complex formed four hydrogen bond interactions with amino acid residues LEU A: 718, CYS A: 797, and ASP A: 855 (Figure 9B). The JAK2‐oridonin complex was deemed stable when the Rg value ranged from 2.18 to 2.22 nm and the RMSD value from 0.30 to 0.35 nm, forming eight hydrogen bond interactions with THR A: 82, ASP A: 292, THR A: 81, GLN A: 79, TRP A: 80, and VAL A: 271, as well as four Alkyl interactions with VAL A: 270, ARG A: 273, and ILE A: 84 (Figure 9C). Lastly, the EGFR‐oridonin complex maintained stability with an Rg value of 2.03 to 2.07 nm and an RMSD value of 0.275 to 0.3 nm. During this stable conformational state, the complex engaged in three hydrogen bond interactions with ASN A: 981, ARG A: 980, and LEU A: 855, along with two Alkyl interactions with VAL A: 863 and LEU A: 983 (Figure 9D).

### 3.6 Cell Viability Detection

**FIGURE 10:**
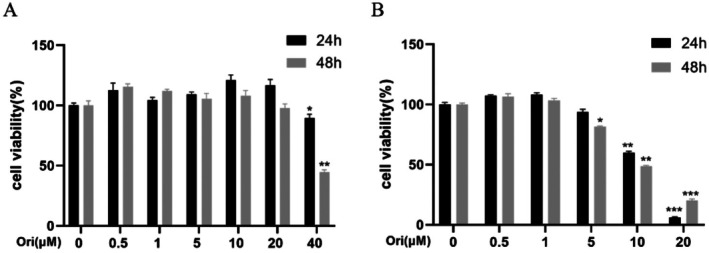
CCK8 assay outcomes of HepG2 (A) and Huh7 (B) cell viability. Results were presented as mean ± SD, with a sample size of 3. *p < 0.05; **p < 0.01; ***p < 0.001 vs. 0 μM.

In comparison to the control group, the oridonin showed minimal impact on the viability of HepG2 cells at 0–20 μM. At a concentration of 40 μM, the average cell survival rate was 89% after 24 h (Figure 10A). After 24 h of administration, the average cell viability of Huh7 remained unchanged at 0–5 μM. At concentrations of 10 μM and 20 μM, the average cell survival rates were 60% and 5.9%, respectively (Figure 10B). As a result, additional validation tests were conducted on Huh7 cells using 1 μM and 7.5 μM of oridonin, and on HepG2 cells using 20 μM and 40 μM.

### 3.7 Effects of Oridonin on HCC Cell‐Cycle and Apoptosis

**FIGURE 11:**
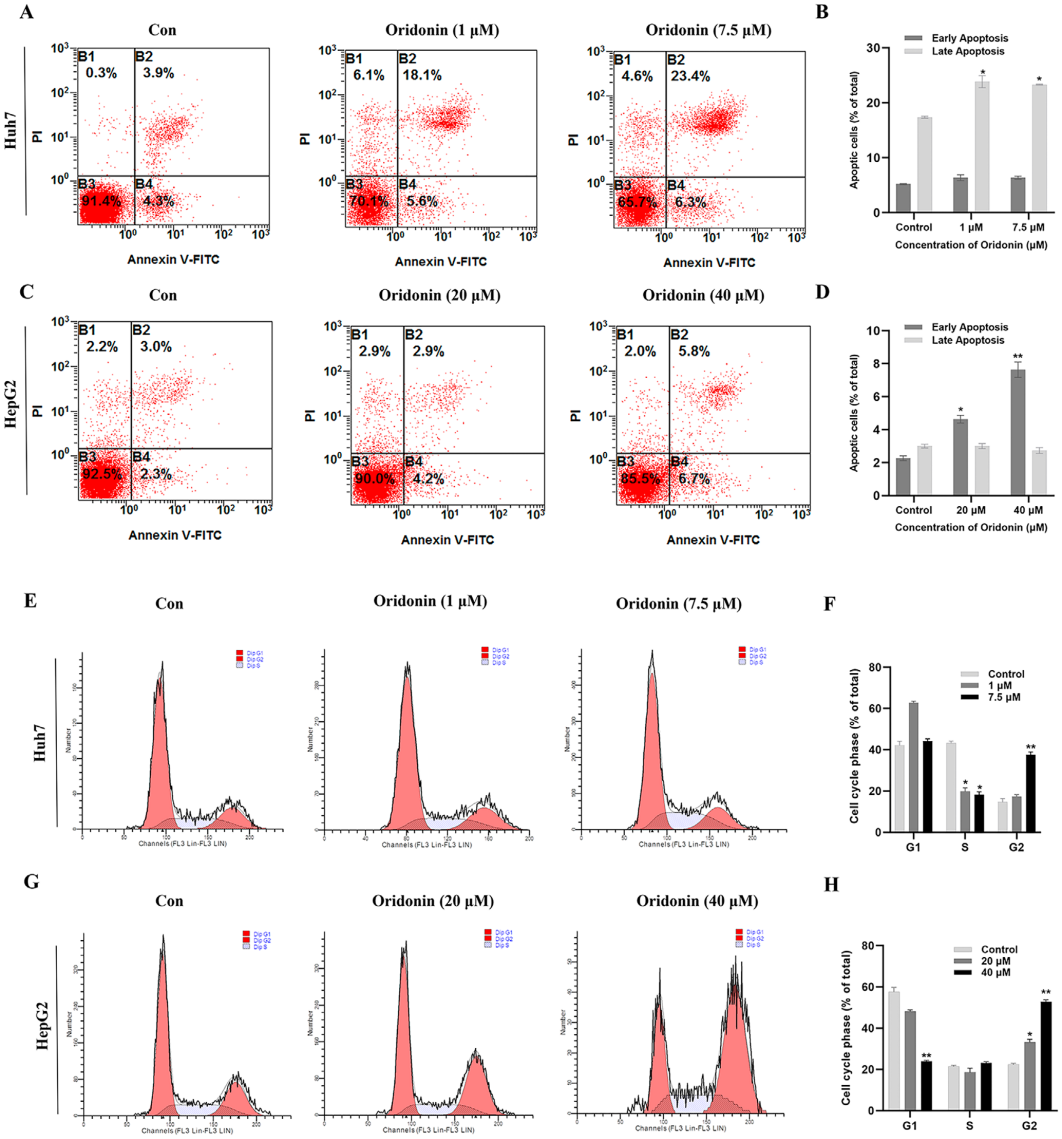
(A–D) Effects of oridonin on HepG2 and Huh7 cells apoptosis. (E–H) Effects of oridonin on the cell cycle of HepG2 and Huh7 cells. Results were presented as mean ± SD, with a sample size of 3. *p < 0.05; **p < 0.01 vs. control group.

Flow cytometry assay was utilized to investigate the impact of oridonin on HCC cell apoptosis and cycle progression. The findings revealed a significant rise in early and late apoptosis of HCC cells following treatment with oridonin in comparison to the control group (*p* < 0.05) (Figure 11A–D). Moreover, the cell cycle assessment demonstrated a reduction in the proportion of cells in G1 and S phases with increasing oridonin concentration, while the number of cells in G2 phase notably increased (*p* < 0.05) (Figure 11E–H). These results indicated that oridonin effectively arrested HCC cells in the G2 phase and stimulated apoptosis.

### 3.8 Oridonin Inhibits mRNA Expression Levels of PIK3R1, AKT1, and EGFR

**FIGURE 12:**
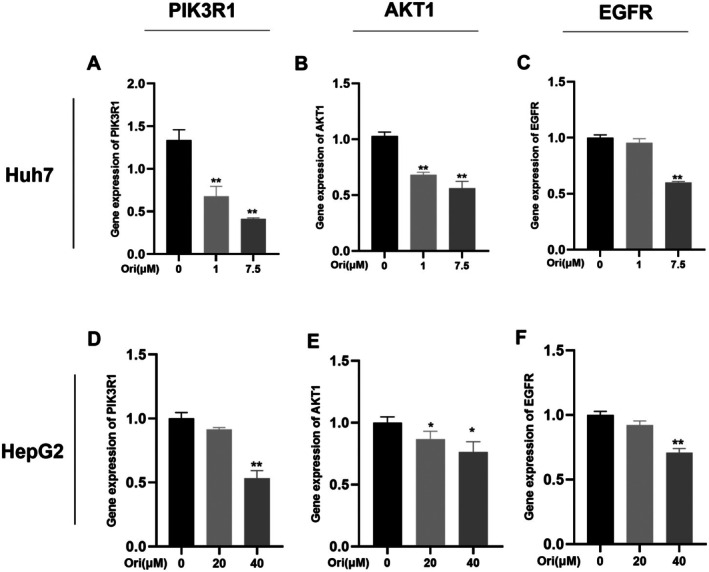
(A–C) The expression level of PIK3R1, AKT1, and EGFR in Huh7 cells. (D–F) The expression level of PIK3R1, AKT1, and EGFR in HepG2 cells. Results were presented as mean ± SD, with a sample size of 3. *p < 0.05; **p < 0.01 vs. control group.

To validate the outcomes of network pharmacology, we conducted an analysis of the principal targets within the EGFR/PI3K/AKT signalling pathway. The qRT‐PCR analysis demonstrated a significant, dose‐dependent downregulation in the mRNA expression levels of PIK3R1, AKT1, and EGFR (Figure 12). Details of the primer sequences utilized in the analysis can be found in Table 2.

**TABLE 2:** Primer sequences detected by qRT‐PCR.

Gene	Forward primer (5′‐3′)	Reverse primer (3′‐5′)
GAPDH	TGACATCAAGAAGGTGGTGAAGCAG	GTGTCGCTGTTGAAGTCAGAGGAG
AKT1	ACTGTCATCGAACGCACCTT	CTCCTCCTCCTCCTGCTTCT
PIK3R1	AGGTGAAGCTCGTGTGTGGA	GAAGACAGGGCTCCACTTCC
EGFR	GGTGAGTGGCTTGTCTGGAA	CCTTACGCCCTTCACTGTGT

### 3.9 Oridonin Suppresses the Protein Expression Levels of EGFR/PI3K/AKT Signalling Pathway

**FIGURE 13:**
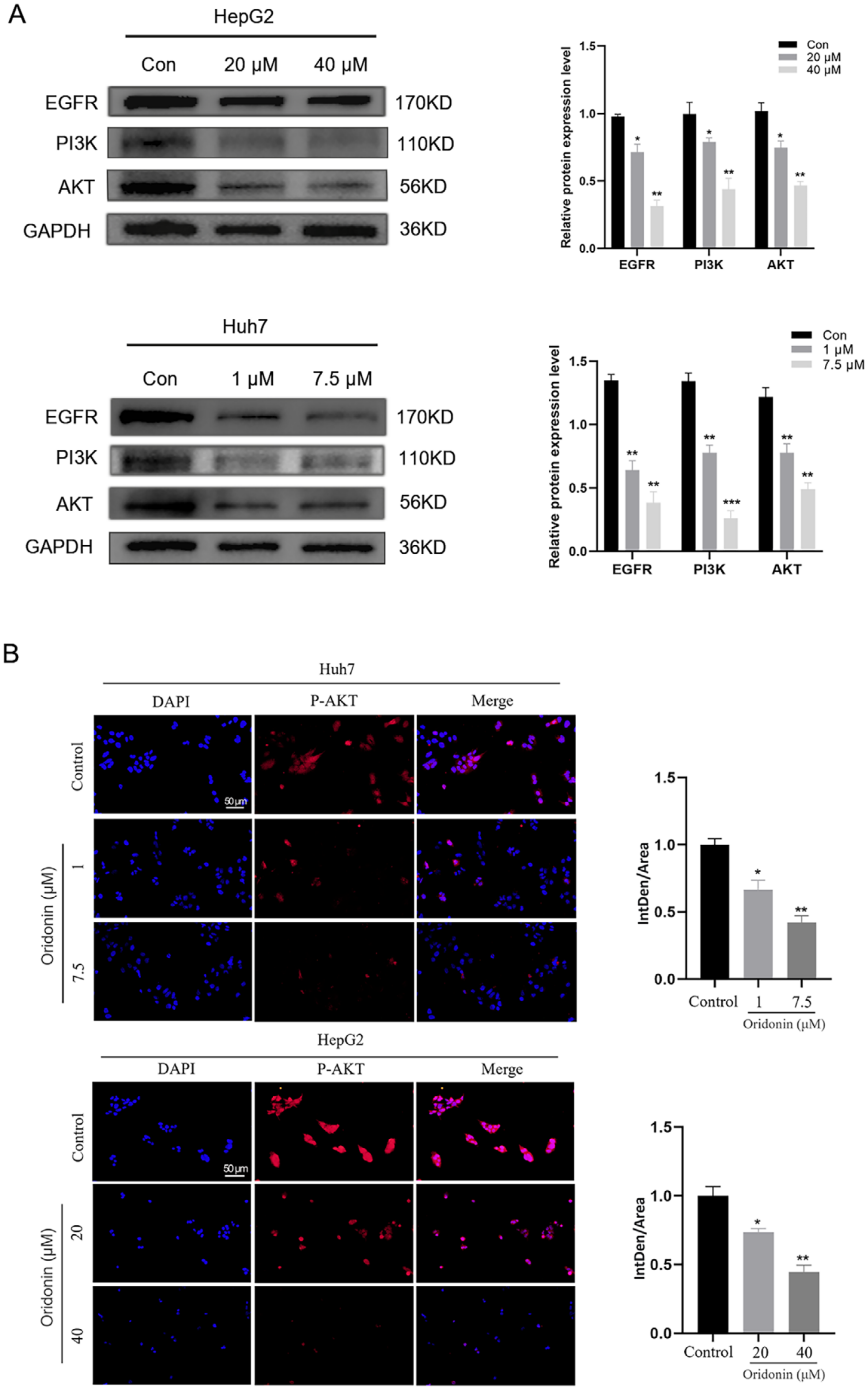
Oridonin inhibits PI3K‐AKT pathway at protein expression level. (A) Western blotting of EGFR, AKT, and PI3K expression in cells. (B) Immunofluorescence of p‐AKT. Results were expressed as the mean ± SD, with a sample size of 3. *p < 0.05; **p < 0.01 vs. control.

Utilizing Western Blotting, we further analysed the expression levels of key proteins involved in the EGFR/PI3K/AKT signalling pathway (Figure 13A). The results demonstrated that, in comparison to the control group, the protein expression levels of PI3K, AKT, and EGFR in HCC cells treated with oridonin showed a significant reduction (*p* < 0.05). The initiation of the PI3K‐AKT pathway relies on AKT protein phosphorylation [34]. Hence, a study was conducted on the levels of p‐AKT expression in the pathway (Figure 13B). The findings revealed a substantial decrease in p‐AKT protein levels in HCC cells exposed to oridonin (*p* < 0.05). These findings suggested that oridonin might inhibit the activation of the EGFR/PI3K/AKT signalling pathway, thereby impeding cell cycle progression and promoting apoptosis.

## 4 Discussion

Advancements in surgical resection, liver transplantation, and chemotherapy drugs have improved the diagnosis and treatment of HCC. However, challenges persist with poor prognosis and high rates of recurrence [35, 36]. Oridonin, the core active constituent derived from Rabdosia rubescens, exhibits potent anti‐tumour activity [37]. Current research on the effects of oridonin on HCC predominantly emphasizes its synergistic interactions with other anti‐cancer agents [38, 39]. Furthermore, a limited number of studies have demonstrated that oridonin can induce apoptosis in HCC cells through the activation of caspase‐3, down‐regulation of Bcl‐2, and up‐regulation of Bax expression, thereby inhibiting cellular proliferation, with most of these studies focusing on single cell types [40, 41]. This research adopts a comprehensive approach by integrating network pharmacology, molecular docking, and MDS as bioinformatics tools, complemented by in vitro validation experiments using HepG2 and Huh7 cell lines.

Through network pharmacology, 273 potential therapeutic targets of oridonin were identified. The top 10 core targets were highlighted, including PIK3R1, AKT1, JAK2, EGFR, SRC, IGF1R, BCL2, TP53, CASP3, and PTPN11. Among them, PIK3R1 encodes the regulatory subunit of PI3K, and research has indicated that reduced expression of PIK3R1 leads to growth inhibition of HCC cells, potentially through causing cell cycle arrest in the G2/M phase and enhancing apoptosis [42, 43]. AKT serves as the core node in the intricate network of signalling pathways, usually over‐activated in cancer, thus affecting the mutation or amplification of upstream regulatory factors [44, 45]. The EGFR is a cell surface receptor that becomes activated upon binding to specific ligands, such as EGF or TGF‐α. Ligand binding induces receptor dimerization, which subsequently activates the intrinsic kinase activity of EGFR. The catalytic domain of the EGFR kinase facilitates the process of transphosphorylation, resulting in the phosphorylation of EGFR itself. The phosphorylated tyrosine residues on the activated EGFR cytoplasmic domain serve as docking sites for proteins containing Src homology 2 domains, such as PI3K, thereby initiating downstream signalling pathways [46, 47, 48]. SRC has been shown to modulate various aspects of tumour cell behaviour such as apoptosis, proliferation, migration, invasion, and metastasis [49]. Analysis conducted using GO suggests that oridonin may possess therapeutic potential in HCC by targeting intracellular receptor signalling pathways, regulating apoptosis signalling pathways, and influencing nuclear receptor activity, protein tyrosine kinase, and other molecular functions. This finding aligns with prior research demonstrating the ability of oridonin to suppress AKT phosphorylation, increase Bax expression, and promote tumour cell apoptosis [50]. Additionally, KEGG enrichment analysis indicates that pathways related to cancer, specifically the PI3K‐AKT signalling pathway and cell apoptosis pathways, are impacted. The overstimulation of PI3K‐AKT is a frequent occurrence in numerous types of cancer forms and controls essential cellular functions such as growth, apoptosis, and proliferation [51, 52]. PI3K facilitates the transformation of PIP2 to PIP3, hence phosphorylating AKT to stimulate the initiation of the PI3K‐AKT signalling pathway [53]. The subsequent components are linked with the cellular cycle, cell death, viability, expansion, and glucose utilization [54]. Oridonin is a small‐molecule compound that may regulate target gene transcription and protein expression through two complementary mechanisms. It may bind to the transcription factors that control the expression of PIK3R1, AKT1, and EGFR, thereby blocking their binding to target gene promoters and reducing transcriptional activity. Alternatively, it may promote the ubiquitination and proteasomal degradation of these target proteins. Notably, the above mechanistic explanations are reasonable inferences based on our current experimental results, rather than conclusive evidence. Further rigorous and in‐depth studies are needed to fully clarify its exact mechanism of action, which we will systematically explore in our future research. Analysis of the docking results indicated a binding energy of below −5 kcal/mol for the central target and oridonin, while AKT1, PIK3R1, EGFR, and JAK2 showed higher binding energies. Further analysis was conducted using various parameters such as RMSD, RMSF, Rg and hydrogen bond number to evaluate the stability of the protein‐ligand complex. The RMSD values remained stable at 0.3 nm without significant fluctuations, while the RMSF values consistently stayed at a low level, indicating good stability of the complex. The presence of an appropriate number of hydrogen bonds and a lower Rg suggested that the structures of the complexes were compact and stable. The free energy landscape map illustrated the conformational stability of the complex throughout the simulation.

Subsequent in vitro experiments revealed that oridonin significantly affects the cell cycle and induces apoptosis in HCC cells. Integrated KEGG analysis results, we validated the expression of key targets within the EGFR/PI3K/AKT signalling pathway. qRT‐PCR and Western Blot analysis revealed that oridonin markedly suppresses the mRNA and protein expression levels of AKT, PI3K, and EGFR. Immunofluorescence further demonstrated that oridonin significantly inhibits the phosphorylation of the AKT protein. Research shows that the EGFR/PI3K/AKT signalling pathway is overactivated in HCC cells. Inhibiting the expression of this pathway results in reduced proliferation and enhanced apoptosis of HCC cells. This is consistent with our research results [55, 56].

It is noteworthy that we ultimately chose EGFR/PI3K/AKT signalling as the focus of this study, as all three are among the top ten core targets, and the results of KEGG enrichment analysis showed that the PI3K/AKT signalling pathway ranked second. Numerous literature reports also indicate that EGFR/PI3K/AKT signalling may be a pathological mechanism of HCC. However, our study also has certain limitations, namely, it only selected EGFR/PI3K/AKT signalling and conducted cell experiments (in vitro), requiring more recent animal experiments (in vivo) to further support the scientific validity of the research conclusions. Our next goal is to further explore other mechanisms of action of oridonin in inhibiting HCC.

## 5 Conclusion

This study used an integrated approach involving network pharmacology, molecular docking, MDS, and in vitro experimental validation; these findings provide supportive evidence that oridonin exerts anti‐HCC effects through the EGFR/PI3K/AKT signalling pathway.

## Author Contributions

**Xiaodi Guo:** software, data curation. **Gangqiang Wang:** methodology, validation. **Shan Miao:** conceptualization, validation. **Long Li:** conceptualization, writing – original draft. **Jin Wang:** visualization, conceptualization. **Jing Zhang:** visualization, investigation. **Xiaopeng Shi:** writing – review and editing, funding acquisition. **Shanbo Ma:** writing – review and editing, supervision. **Yang Sun:** writing – review and editing, supervision.

## Funding

This research was supported by Research Institution, Xijing Hospital (No. LHJJ2024‐YX08).

## Conflicts of Interest

The authors declare no conflicts of interest.

## Data Availability

The corresponding author can provide the data presented in this study upon request.
